# Therapeutic Potential of Cannabidiol Cyclodextrin Complex in Polymeric Micelle and Tetrahydrocurcumin Cyclodextrin Complex Loaded in Hydrogel to Treat Lymphedema

**DOI:** 10.3390/ijms26073428

**Published:** 2025-04-06

**Authors:** Waritorn Srakhao, Titpawan Nakpheng, Mohd Cairul Iqbal Mohd Amin, Teerapol Srichana

**Affiliations:** 1 Department of Pharmaceutical Technology, Faculty of Pharmaceutical Sciences, Prince of Songkla University, Songkhla 90110, Thailand; waritorn.s@psu.ac.th (W.S.); titpawan.n@psu.ac.th (T.N.); 2 Centre for Drug Delivery Technology and Vaccine (CENTRIC), Faculty of Pharmacy, Universiti Kebangsaan Malaysia, Jalan Raja Muda Abdul Aziz, Kuala Lumpur 50300, Wilayah Persekutuan Kuala Lumpur, Malaysia; mciamin@ukm.edu.my; 3 Malaysia Genome and Vaccine Institute (MGVI), Jalan Bangi, Kajang 43000, Selangor, Malaysia

**Keywords:** hydrogel, cannabidiol, tetrahydrocurcumin, hyaluronic acid

## Abstract

Cannabidiol (CBD) and tetrahydrocurcumin (THC) have demonstrated anti-inflammatory activity as well as generating new lymph vessels. We present the formulations and evaluations of CBD and THC loaded in hydrogels for the treatment of lymphedema to promote angiogenesis of lymph vessels and an anti-inflammatory response. Six CBD-THC hydrogel formulations were prepared and evaluated. The hydrodynamic particle sizes were 302.0–545.1 nm and the zeta potentials were from −58.80 to −33.63 mV. The hydrogel pHs were 6.43–6.54. The hydrogel formulations were non-toxic for both CBD (<25 µg/mL) and THC (<12.5 µg/mL). It was observed that high-molecular-weight hyaluronic acid in hydrogel affected collagen production. Hydrogel formulations at 2 µg/mL of CBD and 1 µg/mL of THC induced human dermal lymphatic endothelial cell tube formation. CBD-THC hydrogel formulations showed a notable ability to induce angiogenesis, which suggested its potential effectiveness in promoting new lymphatic vessel formation. Moreover, CBD-THC hydrogels showed anti-inflammatory properties. Further research is needed to ensure these treatments effectively enhance lymphatic repair.

## 1. Introduction

Lymphedema is a common chronic disease affecting approximately 1 in 30 people worldwide. It is an abnormality of the lymphatic vessels or lymph nodes associated with a congenital or acquired disorder [1]. The tissue swelling occurs from protein-rich fluid accumulation and impairment of lymphatic drainage, lymphatic flow, and the capacity of the lymphatic circulation. Primary lymphedema may occur at any phase of life but most commonly appears at puberty. Primary lymphedema is caused by developmental lymphatic vascular anomalies or heredity. The clinical manifestation data of mutations in lymphedema are related to the development and function of the lymphatic system. Mutations of three genes, which are vascular endothelial growth factor receptor 3 (VEGFR-3), forkhead box C2 transcription factor (Foxc2), and SRY-box transcription factor 18 (SOX18), lead to primary lymphedema [2,3]. VEGFR-3 is a key protein in lymphangiogenesis that orchestrates various cellular processes such as proliferation, migration, survival, and maintenance of lymphatic endothelial cells. Its activation by vascular endothelial growth factor C (VEGF-C) and vascular endothelial growth factor D is central to the development and remodeling of lymphatic vessels [4,5]. Foxc2 and SOX18 regulate lymphangiogenesis from the differentiation and proliferation of lymphatic endothelial cells to the maintenance of lymphatic valve integrity [2,6]. Secondary lymphedema is caused more often by filariasis, which is a parasitic disease found particularly in South-East Asia. In addition, cancer or radiation from cancer treatment that damages the lymph vessels or surgery to remove cancer may also cause lymphedema [1].

The angiogenic growth of lymphatic vessels coordinates several biological processes such as cell proliferation, guided migration, differentiation, and cell-to-cell communication [7]. Several research studies of lymphedema treatment focused on angiogenesis of lymphatic vessels due to its role in the functioning of the lymphatic system. Lymphatic drainage is another important factor that causes tissue swelling in lymphedema. Although most research was conducted in vitro and in animals, the positive results are considered to be novel strategies for lymphedema treatment. Angiogenesis is the formation of new capillary vessels, which includes blood vessels and lymphatic vessels. The angiogenic growth of blood-vessel capillaries is controlled by Notch signaling [4,8]. Gradients of matrix-bound vascular endothelial growth factor A and other navigational cues are recognized by specialized endothelial cells at the distal end of each sprout. The transcription factor Prospero homeobox 1 is an important regulator of lymphatic endothelial cell (LEC) differentiation. Sprouting, migration and proliferation of LECs is regulated by VEGF-C and VEGFR-3 [9,10,11].

Interleukin-1 (IL-1) plays a key role in initiating the acute inflammatory response. The IL-1 family includes 11 members with similar or distinct biological effects: IL-1α, IL-1β, IL-18, IL-33, IL-36α, IL-36β, IL-36γ, IL-36Ra, IL-37, IL-38, and the IL-1 receptor antagonist (IL-1Ra). These cytokines are secreted by different cell types, including macrophages, monocytes, dendritic cells, and epithelial cells [12]. Typically, IL-1 is not produced by unstimulated cells in healthy individuals, except for specific cell types such as keratinocytes in the skin, certain epithelial cells, and select central nervous system cells. However, exposure to inflammatory stimuli, infections, or microbial endotoxins triggers a significant increase in IL-1 production by macrophages and various other cells. IL-1β is a key regulator in immune and inflammatory responses. Dysregulated or excessive IL-1 production has been associated with several pathological conditions that include sepsis, rheumatoid arthritis, inflammatory bowel disease, acute and chronic myelogenous leukemia, atherosclerosis, neuronal damage, and age-related disorders [13]. IL-1β is a critical mediator in inflammation and tissue damage in lymphedema. Chronic lymphedema often triggers an inflammatory response characterized by elevated levels of pro-inflammatory cytokines like IL-1β. Moreover, it contributes to fibrosis by promoting the activation of fibroblasts and the deposition of extracellular matrix proteins. Dysregulated IL-1β levels can impair lymphangiogenesis, which worsens fluid retention [14,15].

Hydrogels are soft materials engineered to modulate immune responses through controlled biomolecule release and absorption. Among these, hyaluronic acid (HA) hydrogel is commonly used in drug delivery systems [16]. Moreover, in the lymphatic system, HA serves a crucial role as part of the extracellular matrix that binds with lymphatic vessel endothelial hyaluronan receptor-1 and induces lymphangiogenesis. HA is extremely hydrophilic, biocompatible, biodegradable, and non-immunogenic. Furthermore, it is widely found in the epithelial and connective tissues of vertebrates. The molecular weight (MW) of endogenous HA varies in different parts of the body and typically exists as a high-molecular-weight polymer of over 1000 kDa. However, it can be digested to lower MW fragments in the body by hyaluronidase. HA fragments lower than 100 kDa are effective in stimulating vascular endothelial cell proliferation and migration, as well as angiogenesis. HA fragments higher than 500 kDa are used in wound healing, cosmetics, ophthalmology, and anti-inflammatory applications [17].

Cannabidiol (CBD) [C_21_H_30_O_2_; MW: 314.5 g/mol] is the main phytocannabinoid of *Cannabis sativa* L. which has beneficial pharmacological properties that include anti-inflammatory and antioxidant activities [18]. The activity of CBD is mainly due to the presence of hydroxyl groups in the phenolic ring at the C-10 and C-50 positions, the methyl group at the C-1 position of the cyclohexane ring, and the pentyl chain at the C-30 of the phenolic ring [19]. Tetrahydrocurcumin (THC) [C_21_H_24_O6; MW: 372.4 g/mol] is a candidate metabolite of curcumin. The chemical structure of THC is similar to curcumin; however, it lacks double bonds in the central seven-carbon chain within the molecule. In addition, THC is colorless and has more hydrophilic activity than curcumin. However, THC has biological activities similar to curcumin, such as antioxidant, anti-diabetic, anti-hypertensive, and anti-inflammatory effects [20,21]. Recent research demonstrated that THC has a therapeutic potential in various disorders such as hyperalgesia [22], hypercholesterolemia [23], neurotoxicity syndrome [22], and necrosis [24]. Sangartit et al. (2014) found that THC increased arterial stiffness and vascular remodeling in mice [25].

The objectives of this research were to formulate CBD-THC hydrogels and evaluate their in vitro potentials in treating lymphedema. We employed human dermal lymphatic endothelial cells (HDLECs) to evaluate the effectiveness of CBD-THC hydrogel in collagen production, anti-inflammation, and lymphangiogenesis.

## 2. Results

### 2.1. Drug Content of THC and CBD in HA-Based Hydrogel Containing the CBD-β-CD-PM and THC-Me-β-CD Formulations by HPLC

**Figure 1:**
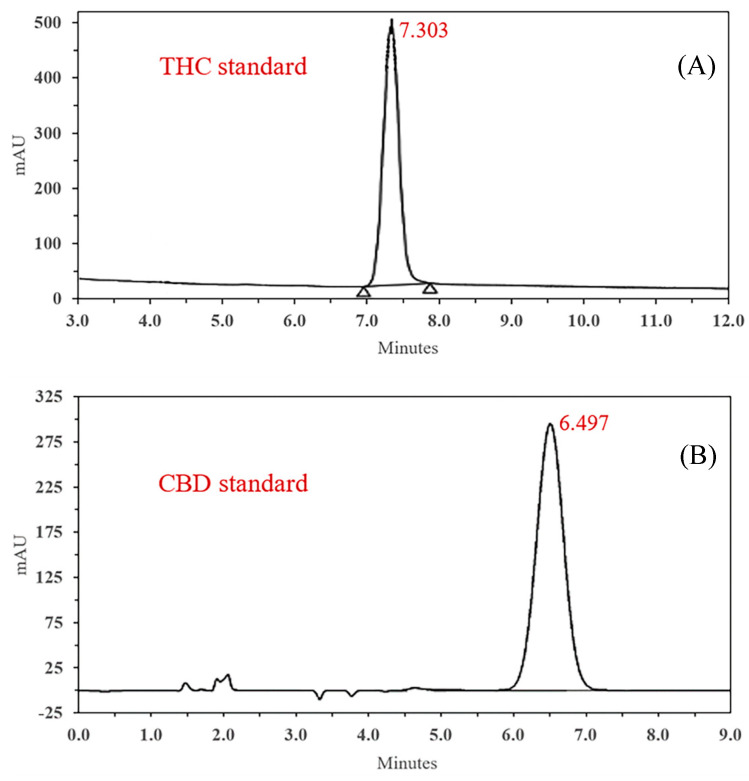
Chromatograms of THC standard (**A**) and CBD standard (**B**).

From the chromatograms of the THC standard solution and CBD standard solution shown in Figure 1, it is evident that under the proposed chromatographic conditions, complete separation of THC was achieved with a retention time of 7.303 min. Furthermore, in the chromatogram of the THC solution, there were no interfering peaks at the retention times of the investigated peaks (Figure 1A). Similarly, in the chromatogram of CBD (Figure 1B), a chromatogram of the CBD standard was obtained with no interfering peaks at a retention time of 6.497 min. The drug content of CBD in CBD-β-CD-PM was 98%, and the drug content of THC in THC-Me-β-CD was 95% (Table 1).

### 2.2. Particle Size Distribution and pH

Considering the specific requirements for injection of lymphedema treatment, the particle size cutoff values were set at ≤500 nm, with an acceptable PDI range of ≤0.5, zeta potential in the range of <−30 mV or >30 mV, and a pH range of 6.5–7.4 [26]. Table 1 shows that the particle sizes of the THC hydrogel formulations (T-1A, T-1B, T-2A, T-2B, T-3A, and T-3B) ranged from 568.20 ± 73.27 nm to 1467 ± 396.19 nm, which exceeded acceptable values and displayed a broad distribution and indicated variability that may affect the hydrogel consistency in terms of particle size. In the CBD hydrogel formulations (C-1A, C-1B, C-2A, C-2B, C-3A, and C-3B), the diameters ranged from 143.13 ± 2.33 nm to 246.33 ± 43.76 nm. The PDI of this group ranged from 0.41 ± 0.01 to 0.54 ± 0.13, which indicated more homogeneous formulations in the CBD hydrogel. The CBD-THC hydrogel formulations (M-1A, M-1B, M-2A, M-2B, M-3A, and M-3B) exhibited average diameters from 302.03 ± 41.10 nm to 545.07 ± 34.11 nm, and the PDI values ranged from 0.42 ± 0.07 to 0.66 ± 0.01. Both the CBD and CBD-THC hydrogels demonstrated sizes within the acceptable range for certain applications, although some formulations, such as M-1B, displayed a broad distribution with a PDI > 0.5, which indicated size variations. The results of the zeta potential tests were as follows: (1) from −62.63 ± 1.69 mV to −37.27 ± 1.21 mV for the THC hydrogel formulations; (2) −56.43 ± 4.77 mV to −27.80 ± 4.14 mV for the CBD hydrogel formulation; and (3) −58.80 ± 1.47 mV to −34.97 ± 1.88 mV for the CBD-THC hydrogel formulations. All formulations of CBD-THC hydrogels and THC hydrogels had zeta potentials from −30 to 30 mV. The formulations of C-2A and C-3A were slightly out of acceptable ranges. However, the CBD-THC hydrogel formulations showed no aggregation, which indicated good dispersion. The results suggested that the THC hydrogel formulations need further improvement to meet the acceptable criteria for injection. The CBD hydrogel and CBD-THC hydrogel formulations generally met acceptable criteria for particle size (≤500 nm), PDI index (≤0.5), zeta potential (<−30 mV or >30 mV), and pH (6.5–7.4). However, some formulations exhibited broader size distribution (M-1B), slight variations in size (C-1A, M-1A, M-1B, and M-2B), and zeta potentials lower than −30 mV (C-2A and C-3A), which may affect consistency and performance. These need optimization to ensure uniform size and optimal zeta potential. Nevertheless, we have chosen all CBD-THC hydrogel formulations (M-1A, M-1B, M-2A, M-2B, M-3A, and M-3B) for testing in the next step.

### 2.3. Gelation and Viscosity of Hydrogel Formulations

**Figure 2:**
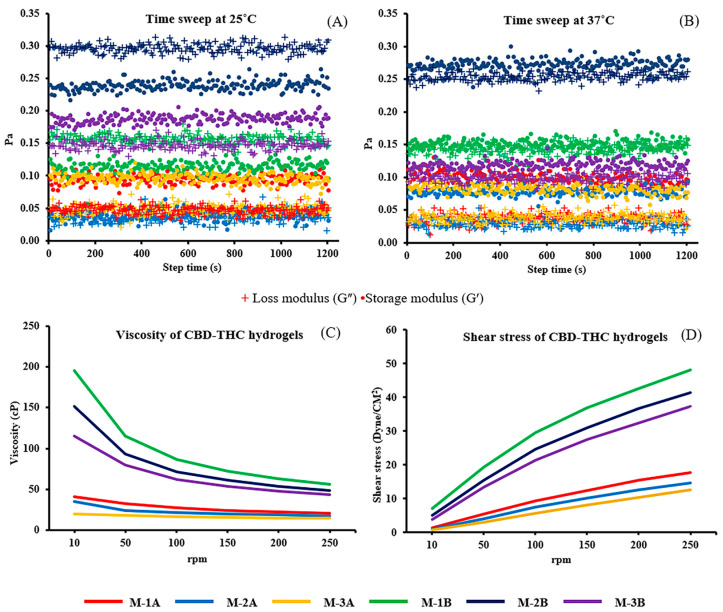
Gelation and viscosity of M-1A, M-1B, M-2A, M-2B, M-3A, and M-3B hydrogel formulations. (**A**,**B**) shows comparison of loss modulus (G″, +) and storage modulus (G′, •). The gelation under time sweeps 25 °C (**A**) 37 °C (**B**). Viscosity (**C**) and shear stress (**D**) of M-1A (red), M-1B (green), M-2A (blue), M-2B (black), M-3A (orange), and M-3B (violet) under defined rates.

Rheology is commonly used to characterize the mechanical properties of hydrogels, especially their gelation and cure time, which directly influence their performance as an injectable. The storage modulus (G′) and loss modulus (G″) indicate the elastic (solid-like) and viscous (liquid-like) behaviors, respectively, which are crucial for application as an injectable biomaterial. Rheology data for the six CBD-THC hydrogel formulations (M-1A, M-1B, M-2A, M-2B, M-3A, and M-3B) that underwent a time sweep test at two different temperatures (25 °C and 37 °C) are shown in Figure 2. The time sweep data indicate how the storage (elastic) modulus of the six CBD-THC hydrogels changed over time at the two temperatures. Generally, the modulus at 37 °C (which is closer to body temperature) was higher compared to 25 °C, which suggests that the gels become stiffer at body temperature. This is a desirable property for injectable hydrogels that need to maintain their structure and mechanical integrity within the body [27,28,29]. The M-1A had no cross-over point of the modulus, which indicated that the material did not change from sol to gel within the time frame observed. The results were similar for both temperatures. This suggested a consistent viscoelastic behavior that makes it predictable in both temperatures. The M-1B also had no cross-over point of the modulus, and at 25 °C, viscous-like behavior dominated (G” > G′), which indicated it behaved more like a liquid. The behavior remained similar at 37 °C. The M-2A had no cross-over point of the modulus, which meant that the transition happened gradually without a clear phase shift, and at 37 °C, solid-like behavior dominated (G′ > G”), which indicated a more gel-like structure that could be beneficial for maintaining integrity after injection. The M-2B also had no cross-over point of the modulus, and at 25 °C, viscous-like behavior dominated (G” > G′), and at 37 °C, viscoelastic-like behavior dominated (G′ > G”), which is ideal for forming a stable gel post-injection. The M-3A had no cross-over point of the modulus. At both temperatures, gel-like behavior dominated (G′ > G″), and the modulus showed similar values. This consistency suggested that it maintained a stable gel state regardless of temperature, which could be advantageous for applications where temperature fluctuations occur. The M-2B had no cross-over point of the modulus, and at both temperatures, gel-like behavior dominated (G′ > G″), which was similar to M-3A. This indicated a robust gel structure that is stable across the temperature range. These results revealed that the higher moduli at 37 °C for some formulations (M-2B and M-3A) suggested that these hydrogels stiffen at body temperature, which is beneficial for maintaining mechanical integrity after injection. Formulations like M-2A and M-3A, for which gel-like behaviors were found at 37 °C, are likely to form stable gels in the body that make them suitable for applications that require prolonged stability. Moreover, the M-1A formulation had consistent behavior across temperatures that could be useful in applications where the hydrogel needs to perform similarly in different environments.

The viscosity data of the six hydrogel formulations in the CBD-THC hydrogels (M-1A, M-1B, M-2A, M-2B, M-3A, and M-3B) under rotation speeds of 10, 50, 100, 150, 200, and 250 rpm are summarized in Figure 2C,D.

As the rotation speed increased from 10 to 250 rpm, the viscosity of M-1B hydrogels decreased from 195.60 to 56.68 cP, that of M-2B hydrogels decreased from 151.8 to 48.82 cP, and that of M-3B hydrogels decreased from 115.20 to 44.02 cP. Similarly, the viscosity of M-1A decreased from 41.40 to 20.66 cP, that of M-2A decreased from 35.40 to 17.48 cP, and that of M-3A decreased from 19.80 to 15.0 cP. The highest viscosity was observed in the M-1B formulation, while the lowest viscosity was noted in the M-3A formulation.

On the other hand, as the rotation speed increased from 10 to 250 rpm, shear stress showed a direct correlation with viscosity. These results revealed that all formulations exhibited shear-thinning behavior with viscosities that decreased as the rates of shear increased. The M-1B formulation, which had the highest viscosity, may be retained longer at the affected site, while the M-3A formulation, which had the lowest viscosity, would likely be easier to inject but may diffuse more quickly within the tissues [30].

### 2.4. Morphology of the Hydrogel

**Figure 3:**
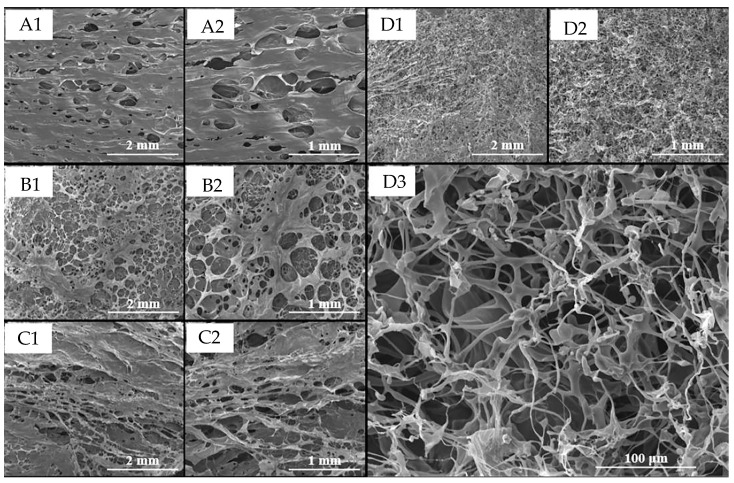
SEM micrographs of the microporous network of blank hydrogels (**A1**,**A2**), THC-hydrogel (**B1**,**B2**), CBD-hydrogel (**C1**,**C2**), and CBD-THC-hydrogel (**D1**–**D3**). The magnification of (**A1**,**B1**,**C1**,**D1**) at 25× (the scale bar is 2 mm). The magnification of (**A2**,**B2**,**C2**,**D2**) at 50× (the length bar is 1 mm), and the magnification of (**D3**) at 350× (the scale bar is 100 µm).

We selected hydrogel formulations T-3B, C-3B, and M-3B as representatives for the THC, CBD, and THC-CBD hydrogel formulation groups, respectively, to understand the dimensional structure of the void spaces within the hydrogel matrix. The HA hydrogel of 3B was used as a blank. All samples underwent freeze-drying before testing. The porosity results of the hydrogels are depicted in Figure 3. The matrices of the blank HA hydrogel, which was formed without THC and CBD at 25 °C, exhibited an average pore size of 283.6 ± 70.6 μm. Hydrogel formulations T-3B, C-3B, and M-3B displayed average pore sizes of 238.9 ± 26.2 μm, 162.6 ± 45.9 μm, and 32.02 ± 6.12 μm, respectively. This indicated that the morphology of the hydrogels underwent significant changes when THC, or CBD, or both were added to the blank hydrogel. All hydrogel formulations were macroporous, but notable differences could be observed. The sizes of most pores in both the T-3B and C-3B gels exceeded 50 μm. However, the polymeric matrix of T-3B was even, while the polymeric matrix of C-3B was uneven, which exhibited a multitude of small and large holes. Moreover, when comparing T-3B with the blank, the morphologies of the polymeric matrices were similar, but the number of macropores increased with the addition of the THC complex. Recent research suggests that the freeze-drying process can generate a porous structure in HA hydrogels with an average pore size that ranges from 160 to 270 μm, which makes them suitable temporary sites for angiogenesis [31]. Furthermore, hydrogels rapidly form a self-healing microporous adhesive scaffold with a pore size of 26.9 μm, which is acceptable for candidates for in situ repair of soft tissue [32,33]. Our observations of HA gel morphologies align with these findings. In the case of hydrogel formulation M-3B, the pore size was much smaller than 50 μm and homogenous. However, the polymeric matrices of M-3B differed significantly from the blank hydrogel by appearing wrinkled and rough with numerous small holes. The differences between the M-3B and blank hydrogels were apparent, and the formation of small holes was likely attributed to the addition of THC and CBD.

It is believed that the hydrophilicity of THC and CBD complexes could disrupt the compact gel matrix to behave as water-releasing channels during the freeze-drying process that results in very small pores. However, the reason remains uncertain. On the other hand, it may be considered that the original pore structures are more likely to be retained because the ice in the pores is likely to be removed earlier than the ice immobilized by the polymeric matrix during the freeze-drying procedure. Based on morphology observations, we suggest that the addition of THC and CBD affects the pore size of the hydrogels. The pore size observed using SEM is consistent with the trend of particle sizes observed using a zetasizer, which indicated that the average pore size would be a suitable temporary site for cell proliferation. This alignment between SEM observations and zetasizer measurements supports the idea that the pore structure of the hydrogels is influenced by the presence of THC and CBD, which reinforces the potential suitability of the hydrogel for cell proliferation in a controlled environment.

### 2.5. In Vitro Cytotoxicity and Imaging Cell Viability

**Figure 4:**
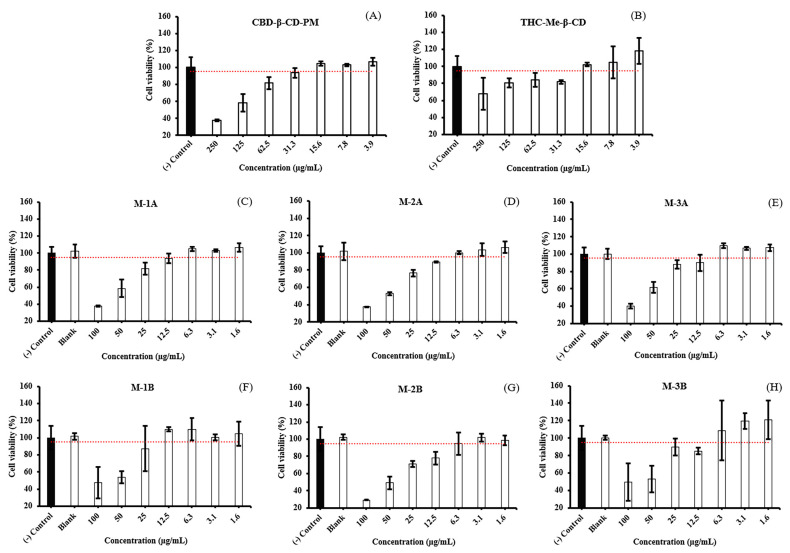
Cell viability of HDLECs cultured on hydrogel formulation treated. (**A**) CBD-β-CD-PM, (**B**) THC-Me-β-CD, (**C**) hydrogel M-1A, (**D**) hydrogel M-2A, (**E**) hydrogel M-3A, (**F**) hydrogel M-1B, (**G**) hydrogel M-2B, (**H**) hydrogel M-3B (mean ± SD, n = 4).

Following cell seeding, attachment to the surfaces occurred within 5 h. Cell viability of the HDLECs was evaluated after 24 h of treatment with different concentrations of blank hydrogel, CBD-β-CD-PM, THC-Me-β-CD, and the six hydrogel formulations (M-1A, M-1B, M-2A, M-2B, M-3A, and M-3B). HDLECs cultured on medium served as a negative control. The concentrations of 62.5 μg/mL CBD-β-CD-PM (Figure 4A), 125 μg/mL THC-Me-β-CD solution (Figure 4B), and blank hydrogel exhibited cell viability exceeding 80%. However, HDLECs treated with the six CBD-THC hydrogel formulations displayed cell viability exceeding 80% at concentrations of CBD-β-CD-PM 12.5 μg/mL and THC-Me-β-CD 6.25 μg/mL (Figure 4C–H). Importantly, at non-toxic concentrations of THC-Me-β-CD below 15.6 μg/mL, a decrease in cell viability was observed. Nevertheless, further studies are necessary to elucidate these activities. In summary, these results indicate that CBD-β-CD-PM and THC-Me-β-CD solutions are non-toxic to HDLECs at concentrations below 62.5 μg/mL. Similarly, the six CBD-THC hydrogel formulations (M-1A, M-1B, M-2A, M-2B, M-3A, and M-3B) are non-toxic at CBD-β-CD-PM concentrations below 12.5 μg/mL and THC-Me-β-CD below 6.25 μg/mL. Consequently, lower concentrations of these formulations were selected for the subsequent testing phase.

### 2.6. Estimation of the Soluble Collagen Produced by HDLECs

Prolonged lymphatic congestion and inflammation can lead to fibrosis, characterized by the excessive formation of fibrous connective tissue, including collagen. This fibrosis contributes to the thickening and scarring of lymphatic vessels that further hinder the normal flow of lymph and exacerbate the symptoms of lymphedema. This current study also investigated the effects of hydrogel formulations on collagen production in HDLECs since understanding how these formulations influence collagen production is critical for developing effective treatments for lymphedema.

**Figure 5:**
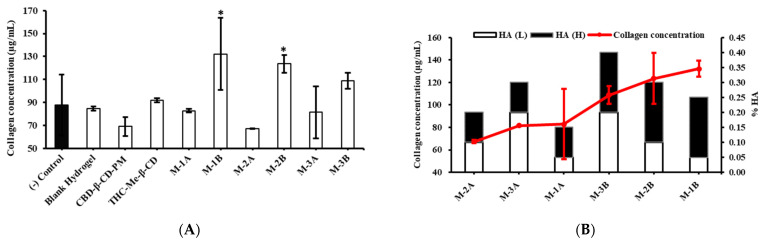
The effect of CBD-THC hydrogel on the collagen production of HDLECs was assessed. (**A**) The collagen concentration in the cell culture medium determined by the optical density (OD). (**B**) Collagen production (red line) and HA percentage (bar graph) (mean ± SD, n = 4). * *p*-value < 0.05.

In this study, we estimated the collagen productivity of HDLECs after treatment with CBD-β-CD-PM, THC-Me-β-CD, and the six CBD-THC hydrogel formulations (M-1A, M-1B, M-2A, M-2B, M-3A, and M-3B) in HDLECs using the Sircol™ assay. The results indicated that hydrogel formulations M-1B, M-2B, and M-3B at a concentration of 2 µg/mL CBD-β-CD-PM promoted collagen production in HDLECs in a concentration-dependent manner. Conversely, collagen production was not higher in cells treated with M-1A, M-2A, and M-3A compared with the control (Figure 5A). Specifically, M-3B and M-2B slightly promoted collagen production (108.84 ± 7.07 μg/mL and 123.58 ± 7.82 μg/mL, respectively), while M-1B significantly enhanced collagen (132.26 ± 31.26 μg/mL). However, given that excessive collagen production exacerbates lymphedema by promoting fibrosis and hindering lymphatic flow, formulations that significantly increase collagen production (M-1B, M-2B, and M-3B) might be counterproductive for treating lymphedema. These formulations should be further investigated.

Figure 5B represents the trends in the amount of collagen and HA percentage. Charts with black bars indicate all formulations containing high MW HA 0.2% (M-1B, M-2B, and M-3B). White bar charts show all formulations containing low MW HA 0.1% (M-1A, M-2A, and M-3A). The formulations with a higher concentration of high MW HA (0.2%) are more effective in promoting collagen production than those with a lower concentration (0.1%). The study highlights that the concentration of high MW HA affects collagen production. Formulations with lower concentrations of high MW HA (0.1%) did not increase collagen production. When considering the concentration of collagen production with total %HA (combination of high and low MW) in Figure 5B, collagen production did not show a significant increase, which indicated that the size and concentration of HA molecules play a crucial role. However, further studies are necessary to explore the effects of various ratios of HA on collagen production and identify formulations that can help manage lymphedema. Studies also need to investigate the underlying mechanisms of how HA affects collagen production in HDLECs which can lead to more targeted treatments.

Formulations that do not promote excessive collagen production are preferable for the treatment of lymphedema. Therefore, M-1B, M-2B, and M-3B formulations with high MW HA at high concentrations are preferable. On the other hand, formulations with lower HA concentrations of low MW HA (such as M-1A, M-2A, and M-3A) could be more beneficial in preventing fibrosis and aiding in effective lymphedema management.

### 2.7. Tube Formation Analysis

**Figure 6:**
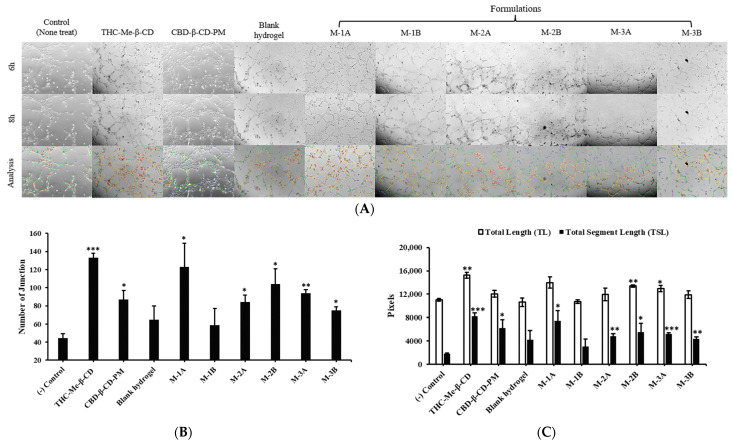
Hydrogel formulation induced HDLEC tube formation. (**A**) HDLECs were incubated in 24-well plates coated with Matrigels and exposed to CBD-β-CD-PM, THC-Me-β-CD, HA-based hydrogel, hydrogel M-1A, M-1B, M-2A, M-2B, M-3A, and M-3B formulation for 6 h and 8 h. Cells were observed using fluorescence microscopy. Branches are in green, segments are in yellow and junctions are in red (**B**) Number of junctions of HDLECs after treatment for 8 h. (**C**) Total length and total segment length of HDLECs after being treated for 8 h (mean ± SD, n = 3). *p*-value, * < 0.05, ** < 0.01, and *** < 0.001.

The formation of capillary-like tubules is a unique property of endothelial cells [34]. We investigated whether HA hydrogels are capable of promoting angiogenesis in HDLECs in vitro using the Matrigel assay. Enhancing angiogenesis could potentially help in treating lymphedema by improving blood and lymphatic flow, thus aiding in fluid drainage and reducing swelling. The Angiogenesis Analyzer tool in the Image J software version 1.54d, which focuses on the structural analysis of capillary-like tube networks, was used to perform a tube formation assay. The analysis of branch and segment content from ImageJ version 1.54d was according to the following definitions. Branches were networks, which linked one junction to one extremity in green. Segments were networks in yellow lines. The connection between segment and branch is the red junction. The number of junctions, the total length, and the total segment length are common parameters of comparative measurement [35]. In Figure 6B, the number of junctions of HDLECs that were not treated using only the control was estimated to be 44 ± 5. However, the number of junctions of HDLECs using the Matrigel assay after treatment with six hydrogel formulations ranged from 59 ± 18 to 123 ± 26, and the number of junctions of HDLECs treated with THC-Me-β-CD and CBD-β-CD-PM were about 133 ± 5 and 87 ± 10, respectively. These results were similar to the total length and total segment length of the HDLECs in Figure 6C. The total length of the untreated cells was 11,013 ± 220 pixels. The total lengths of the cells treated with six CBD-THC hydrogel formulations ranged from 10,722 ± 310 to 13,973 ± 962 pixels. And the total lengths of HDLECs treated with THC-Me-β-CD and CBD-β-CD-PM were 15,232 ± 519 pixels and 12,043 ± 591 pixels, respectively. The total segment length of the cells that were not treated was 1775 ± 124 pixels. The total segment lengths of the cells treated with the 6 CBD-THC hydrogel formulations ranged from 3015 ± 1313 to 7419 ± 1728 pixels. The total segment lengths of HDLECs treated with THC-Me-β-CD and CBD-β-CD-PM were 8224 ± 545 and 6186 ± 1437 pixels, respectively. The number of junctions of HDLECs after treatment with the THC-Me-β-CD solution was the highest. That indicated that THC-Me-β-CD induced the intersections or branching points formed by HDLECs during the process of angiogenesis. However, HDLECs treated with the CBD-β-CD-PM solution induced the process of angiogenesis less than THC-Me-β-CD. The total length of HDLECs refers to the entire length of all the tubes formed by HDLECs by assessing the overall extent of the tube network. Similarly, the total segment length of HDLECs refers to the cumulative straight segments or branches between junctions in the tube-like structures that were formed by HDLECs. The results indicated that five hydrogel formulations (M-1B, M-2A, M-2B, M-3A, and M-3B) slightly induced HDLECs tube formation after 6 h and 8 h compared with the control. However, while promoting angiogenesis, it is important to balance the risk of excessive collagen production which can lead to fibrosis. Formulations, such as M-1B, M-2B, and M-3B, that significantly increase collagen production may need to be optimized or combined with anti-fibrotic strategies to prevent worsening of lymphedema. Moreover, the M-1A hydrogel formulation showed a notable ability to induce angiogenesis among the hydrogel formulations, which suggested its potential effectiveness in promoting new lymphatic vessel formation. In addition, HDLECs exposed to 2 µg/mL of THC-Me-β-CD markedly enhanced angiogenesis and significantly increased the number of junctions, total length, and total segment length of tube-like structures formed by HDLECs after 6 h and 8 h.

Therefore, the data suggest that THC-Me-β-CD demonstrated the highest angiogenic potential, which makes it a promising candidate for further investigation in lymphedema treatment. Its ability to significantly increase tube formation suggests it could enhance vascular and lymphatic repair. Furthermore, the hydrogel M-1A formulation showed notable angiogenic effects that make it a potential formulation to promote tissue repair and improving lymphatic function in vitro.

### 2.8. Anti-Inflammatory Assay

The levels of IL-1β in the supernatant of THP-1 cells after incubation with CBD-β-CD-PM (15 μg/mL), THC-Me-β-CD (15 μg/mL), blank hydrogel, and the six CBD-THC hydrogel formulations (15 μg/mL) are shown in Table 2. The level of IL-1β in the negative control was 3.99 ± 0.15 pg. This differed from the supernatant of the THP-1 cells incubated with 1 μg/mL of LPS in which the level of IL-1β was 68.18 ± 4.66 pg. This confirmed that 1 μg/mL of LPS successfully activated THP-1 cells compared to the control.

On the other hand, CBD-β-CD-PM (15 μg/mL), THC-Me-β-CD (15 μg/mL), blank hydrogel, and the six CBD-THC hydrogel formulations (15 μg/mL) showed low activated THP-1 cells compared to the LPS-activated group. The levels of IL-1β after incubation with CBD-β-CD-PM, THC-Me-β-CD, and blank hydrogel were 4.85 ± 2.01 pg, 11.85 ± 2.69 pg, and 8.35 ± 3.89 pg, respectively. CBD-THC hydrogel formulations (M-1A to M-3B) showed various levels of IL-1β compared to the LPS activated group, whereas the levels of IL-1β in the THP-1 cell supernatant ranged from 8.09 ± 0.85 to 22.37 ± 1.92 pg. However, M-1A seemed to have had the most pronounced effect in activated IL-1β. The M-1B, M-2A, M-2B, M-3A, and M-3B formulations also demonstrated effects in activated IL-1β levels but to a lesser extent than M-1A.

Table 2 shows the percentage of IL-1β inhibition of the sample in the supernatant of THP-1 cells after incubation with LPS (1 μg/mL) and samples. The percentage of IL-1β inhibition indicates how effectively each treatment reduced the IL-1β level compared to the LPS control. THC-Me-β-CD (15 μg/mL) has a very low inhibition percentage (4.53 ± 2.70%), which indicated it was less effective on its own in reducing IL-1β levels compared to 15 μg/mL CBD-β-CD-PM (11.72 ± 1.68%). All hydrogel formulations (M-1A to M-3B) showed significantly higher IL-1β inhibition compared to CBD or THC alone, which ranged from 56.86 ± 6.10 to 68.97 ± 3.70%. M-2A and M-2B exhibited the highest IL-1β inhibition percentages, which suggested these formulations are the most effective at suppressing the inflammatory response. M-1A, M-1B, M-3A, and M-3B also demonstrated strong inhibition but were slightly less effective compared to M-2A and M-2B.

## 3. Discussion

The development of CBD-THC hydrogels for the treatment of lymphedema presents physicochemical properties suitable for localized administration with a promising ability to promote lymphangiogenesis and modulate anti-inflammation. The below discussion addresses key findings from our study, linking them with prior research and highlighting future research directions. The rheological properties of the CBD-THC hydrogel formulations demonstrated shear-thinning behavior, which is a critical characteristic for injectable biomaterials, as it ensures ease of administration through a syringe while maintaining structural integrity post-injection. Our results showed that viscosity decreased with increasing shear rate, with M-1B exhibiting the highest viscosity among all formulations. The storage modulus (G′) was higher than the loss modulus (G″) at body temperature for most formulations, suggesting their ability to form stable gels upon injection. This property supports their potential use for localized drug delivery in lymphedema, where sustained retention and controlled drug release are essential. The ability to maintain a stable gel structure at 37 °C suggests that these formulations could retain therapeutic function at physiological conditions. The mechanical properties observed in our study align with other studies on HA-based injectable hydrogels designed for drug delivery and tissue engineering applications [36,37]. However, additional in vivo testing is necessary to evaluate how these rheological properties translate to retention and distribution after injection into lymphedematous tissues. Our study revealed that high MW HA significantly influenced collagen production in HDLECs. The formulations containing high MW HA (0.2%) induced greater collagen production compared to those with low MW HA (0.1%). While collagen is essential for tissue repair, excessive collagen production may contribute to fibrosis, which can exacerbate the progression of lymphedema by reducing tissue flexibility and further impeding lymphatic flow [38]. Prior studies have suggested that high MW HA (>500 kDa) is associated with increased fibroblast activity and extracellular matrix deposition [39]. Therefore, formulations with lower concentration of high MW HA may be preferable to avoid unintended fibrotic effects while maintaining lymphatic repair potential. Consequently, for therapeutic application in lymphedema, an optimal balance of HA concentration is required. Further studies should explore modifications in HA composition to minimize fibrosis risk while maintaining hydrogel stability.

Angiogenesis is a critical factor in the treatment of lymphedema, as it promotes the formation of new lymphatic vessels, thereby improving fluid drainage and reducing swelling. The tube formation assay demonstrated that all CBD-THC hydrogel formulations induced tube formation in HDLECs, M-1A showing the most significant pro-angiogenic activity. Additionally, THC-Me-β-CD at 2 µg/mL markedly enhanced angiogenesis, indicating its potential as a key contributor to lymphatic vessel regeneration. These results align with previous research indicating that cannabinoids and curcumin derivatives can modulate endothelial function and vascular growth. The ability of THC to enhance tube formation may be attributed to its structural similarity to curcumin, which has been shown to activate VEGFR-3 and promote lymphangiogenesis [40]. Prior studies have also indicated that cannabinoids can regulate angiogenesis through endothelial cell proliferation and migration [41]. While angiogenesis is beneficial for improving lymphatic drainage, excessive vascularization can lead to unwanted complications such as abnormal vessel permeability and inflammation. Thus, future investigations should explore dose-dependent effects and potential combinatory approaches to optimize the balance between vessel formation and vascular integrity or investigate the molecular mechanisms underlying these effects, such as their interactions with VEGFR-3, Foxc2, and SOX18 signaling pathways.

Inflammation plays a pivotal role in the pathophysiology of lymphedema, with IL-1β being a key mediator of chronic inflammation and fibrosis. Our study demonstrated that CBD-THC hydrogels significantly reduced IL-1β levels in LPS-activated THP-1 cells, with M-2A and M-2B showing the highest inhibition of IL-1β. This suggests that CBD and THC synergistically modulate inflammatory pathways, potentially offering therapeutic advantages over single-drug treatments.

CBD has been extensively studied for its immunomodulatory and anti-inflammatory effects, primarily through its interaction with the cannabinoid receptors CB1 and CB2, as well as peroxisome proliferator-activated receptors (PPARs) [42]. THC, on the other hand, has demonstrated effects in reducing oxidative stress and inhibiting pro-inflammatory cytokines [43]. The observed reduction in IL-1β levels suggests that CBD-THC hydrogel formulations could serve as an alternative or complementary approach to corticosteroids for managing inflammation in lymphedema. Further in vivo studies are necessary to validate these findings and determine their long-term immunomodulatory effects.

While this study establishes the foundational potential of CBD-THC hydrogels for lymphedema treatment, further research is warranted to refine and optimize these formulations. Optimization of HA Concentrations: A more detailed investigation into the molecular weight distribution of HA is necessary to prevent fibrosis while maintaining hydrogel stability. In vivo Evaluation: Animal models of lymphedema should be employed to assess the efficacy, biodistribution, and pharmacokinetics of the hydrogel formulations. Mechanistic Studies: Investigating the molecular pathways involved in CBD-THC-mediated angiogenesis and inflammation regulation will provide deeper insights into their therapeutic potential. Long-Term Stability and Drug Release: The stability and release kinetics of CBD and THC from the hydrogel should be evaluated under physiological conditions to ensure sustained therapeutic effects.

## 4. Materials and Methods

### 4.1. Materials

THC with ≥95% purity (HPLC), low MW HA (10 kDa), high MW HA (1000 kDa), and propylene glycol (PG) were purchased from MySkinRecipes^®^ (MySkinRecipes^®^, Bangkok, Thailand). CBD with 99% purity (HPLC) was obtained from AVS Innovation (Bangkok, Thailand). Poloxamer 407 (Kolliphor P407) was supplied by BASF Canada Inc. (Mississauga, ON, Canada). Methyl-β-cyclodextrin (Me-β-CD) was purchased from Honeywell Fluka™ (Fisher Scientific, Pittsburgh, PA, USA). Chlorobutanol BP was obtained from Srichand United Dispensary Co., Ltd. (Bangkok, Thailand). Ethanol (99%) and acetonitrile were purchased from RCI Labscan (Bangkok, Thailand). Trifluoroacetic acid (TFA) and β-CD were purchased from Sigma-Aldrich (Sigma-Aldrich, Inc., St. Louis, MO, USA). RPMI-1640 medium, fetal bovine serum (FBS), penicillin–streptomycin, 55 mM β-mercaptoethanol, 3-(4,5-dimethylthiazol-2-yl)-2,5-diphenyltetrazolium bromide (MTT), CTS™ Versene™ solution, and dimethyl sulfoxide (DMSO) were purchased from Gibco (Gibco™, LifeTechnologies, Grand Island, NY, USA). The primary HDLECs, endothelial cell basal medium MV2, and growth medium MV2 supplement mix, were purchased from PromoCell (PromoCell, Heidelberg, Germany). Human monocytic cell lines (THP-1) were obtained from ATCC (ATCC, TIB-202, Manassas, VA, USA). The Quantikine^®^ Human IL-1β/IL-1F2 Immunoassay kit (Catalog Number DLB50, R&D Systems, Inc., Minneapolis, MN, USA) was used to measure IL-1β. All other materials were of analytical grade.

### 4.2. CBD-THC Hydrogel Formulation

#### 4.2.1. Preparation of the CBD-β-Cyclodextrin Inclusion Complex (CBD-β-CD)

**Figure 7:**
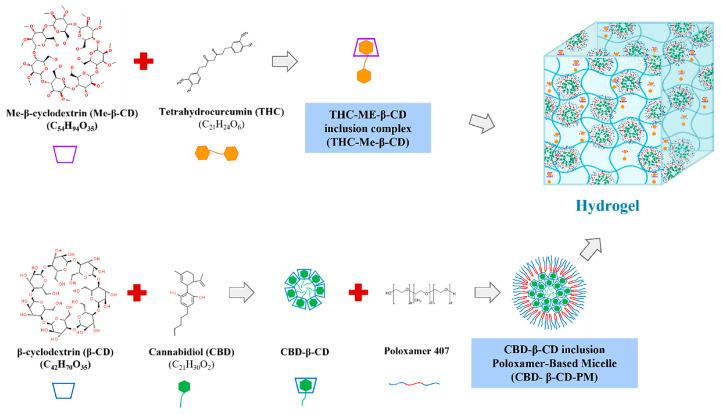
Schematic representation for the fabrication of hydrogel formulation comprises CBD-β-CD-PM and THC-Me-β-CD.

CBD (6.3 g) was dissolved in ethanol (20 mL) while β-CD (22.7 g) was separately dissolved in ultrapure water (20 mL) at 60 °C. Subsequently, the ethanolic CBD solution was transferred slowly to the β-CD solution while stirring using a magnetic stirrer at 300 rpm [44] (Figure 7). The volume was then adjusted to 100 mL using sterile water. The resultant mixture was lyophilized in an FTS Flexi-Dry™ freeze-dryer (FTS Systems, New York, NY, USA) for 42 h. Afterwards, the lyophilized powder (CBD-β-CD inclusion complex) [44] was collected and protected from light by storing in a 50% relative humidity desiccator at 25 °C.

#### 4.2.2. Preparation of the CBD-β-CD Inclusion Poloxamer-Based Micelle (CBD-β-CD-PM)

A quantity of 47.5 mg of the CBD-β-CD inclusion complex was dissolved in 20 mL of purified water. Then, 6.25 mg of poloxamer 407 (PM) was added and stirred until completely dissolved [44]. The volume was adjusted to 100 mL using sterile water (Figure 7).

#### 4.2.3. Preparation of THC-Me-β-CD Inclusion Complex

A 1:1 ratio of THC to Me-β-CD was used in this formulation according to Loron et al., 2021 [45]. Briefly, THC (500 mg) was dissolved in ethanol (50 mL) while the Me-β-CD (1.75 g) was also separately dissolved in ethanol (50 mL). The ethanolic THC solution was then added slowly to the Me-β-CD solution while stirring in a beaker using a magnetic stirrer at 300 rpm until completely mixed. The solution was concentrated by removing part of the ethanol in the mixture using a rotary evaporator (Cole Parmer, Vernon Hills, IL, USA) attached to a vacuum pump and a cooling chamber (CTL 901, USA) at a temperature of 40 °C. Afterwards, the concentrate was lyophilized in an FTS Flexi-Dry™ freeze-dryer for 42 h (Figure 7). The resultant powder was collected into an airtight container and stored in a desiccator protected from light.

#### 4.2.4. HA-Based Hydrogel Containing CBD-β-CD-PM and THC-Me-β-CD

Table 3 shows ingredients and their functions. HA is gelling agent. Chlorobutanol is preservative, propylene glycol is co-dispersion medium. CBD-β-CD-PM and THC-Me-β-CD are active substances. Sterile water for injection is dispersion medium. A blank hydrogel was prepared by dispersing either 0.1 g or 0.2 g of high MW HA (Table 4) in 30 mL of cold sterile water while stirring with a magnetic stirrer at 300 rpm. Then, 2 mL of PG was added into the HA suspension and stirred until fully hydrated. Each of 0.05 g, 0.1 g, and 0.2 g of low MW HA was separately dissolved in 10 mL sterile water in a beaker and stirred using a magnetic stirrer until fully dissolved. The low MW HA solution and high MW HA solutions were mixed together, and 0.5 g of chlorobutanol was added to the mixed hydrogel and stirred at 25 °C to form a blank HA hydrogel. Hydrogels containing different concentrations of THC and CBD were prepared by dissolving THC-Me-β-CD powder or CBD-β-CD-PM powder, respectively (Table 4), in sterile water (10 mL) and mixed until homogeneity. The CBD-β-CD-PM solution and THC-Me-β-CD solution were added to the blank HA-base hydrogel and volume adjusted to 100 mL using sterile water (Figure 7, Table 4). 

### 4.3. Evaluation of the CBD-THC Hydrogel Formulations

#### 4.3.1. Particle Size Analysis

The particle size, polydispersity index (PDI), and zeta potential of the CBD-β-CD-PM, THC-Me-β-CD, and hydrogel formulations were determined using a Zetasizer Nano ZS (Malvern Instruments, Malvern, UK). The running conditions were set at ambient temperature (25 °C) and at a wavelength of 633 nm with a backscattering angle of 173°. The CBD refractive index was 1.540, and the THC refractive index was 1.575. Ultrapure water was used as a dispersant with a refractive index of 1.33. Triplicate determinations were carried out, and the average values of particle size, PDI, and zeta potential were recorded.

#### 4.3.2. Rheology and pH

The gelation behavior of the hydrogel was analyzed using a Discovery Hybrid Rheometer (Discovery Hybrid HR20, TA Instruments, New Castle, DE, USA). The hydrogel formulations were evaluated by comparing the storage modulus (G′) and loss modulus (G″). During injection, the hydrogel should exhibit a higher loss modulus (G″ > G′) to prevent premature gelling. After injection, the storage modulus should increase (G′ > G″) to facilitate gelling and form a stable structure [46]. THC-CBD hydrogel formulations were loaded onto a parallel plate setup (ϴ = 40 mm) with a Peltier-controlled stainless-steel plate and a solvent trap. Oscillatory mode experiments were conducted at 25 °C and 37 °C with a maximum strain of 1% and an angular frequency of 10 rad/s over a duration of 1200 s. The viscosity of the hydrogel preparations was determined using a rotational viscometer (Brookfield DV-III Ultra Rheometer; Brookfield Engineering Laboratories Inc., Middleborough, MA, USA). Briefly, 15 mL of hydrogel was placed in a tube. A spindle number 32 (Brookfield Engineering Laboratories Inc., Middleborough, MA, USA) was dipped in the hydrogel and rotated at 10, 50, 100, 150, 200, and 250 rpm at 25 °C. Each measurement was repeated five times and the average hydrogel viscosity calculated. The pH was determined using a digital pH meter (Eutech pH700, Singapore). The pH meter was calibrated before each use with buffer solutions of pH 4.01, 7.00, and 10.01. Each measurement was repeated in triplicate and the average value of hydrogel pH was calculated.

#### 4.3.3. CBD and THC Analysis

Quantitative measurements of the CBD and THC complex and CBD and THC in the extracted samples were carried out using a high-performance liquid chromatography (HPLC) SpectraSYSTEM™ HPLC system (ThermoFisher Scientific, Waltham, MA, USA). The CBD analysis was performed on a C18 reverse-phase column (150 mm × 4.6 mm, 5 μm) at 25 °C. The mobile phase consisted of acetonitrile/ultrapure water (70:30 *v*/*v*). The mobile phase was used to dissolve the standard and sample solutions. HPLC solvents (mobile phase and MilliQ water), and sample solutions were filtered using a 0.45 μm membrane filtration system (Millipore^®^, EMD Millipore Corporation, Danvers, MA, USA) before injection. The run time for the analysis was 10 min and the injection volume was 50 μL. The flow rate was set at 1.0 mL/min and the wavelength of detection was 207 nm. The HPLC analytical system is very specific to CBD with a 6.5 min retention time [47].

The THC separations were performed on a C18 reverse-phase column (150 mm × 4.6 mm, 5 μm) at 25 °C. The mobile phase consisted of acetonitrile:0.01 mM TFA (30:70 *v*/*v*) adjusted to a pH of 3.0 ± 0.5. The run time for the analysis was 10 min, and 50 μL was the injection volume. The flow rate was set at 1.2 mL/min and the wavelength of detection was 280 nm. The HPLC analytical system was very specific to THC with a 7.3 min retention time [48]. The acquired data were processed using Chromquest™ software version 5.0.

#### 4.3.4. Evaluation of CBD and THC Content of CBD-THC Hydrogels

HPLC was employed to determine the CBD and THC content of CBD-β-CD-PM, THC-Me-β-CD, and the hydrogel formulations. Samples were extracted before analysis. Ethyl acetate was used for the extraction. It was added to the liquid formulations at a 1:1 ratio and mixed by vortexing for 2 min. The solvent layer was allowed to separate for 5 min. The organic solvent phase was then removed. This process of adding ethyl acetate was repeated three times. Samples were dried using a stream of nitrogen gas and the dried residue obtained was stored at 4 °C. Dried residues were re-dissolved at 25 °C for the HPLC analysis using the mobile phase solution and analyzed using previously described HPLC methods.

#### 4.3.5. Morphology of HA-Based Hydrogels Containing CBD-β-CD-PM and THC-Me-β-CD by Scanning Electron Microscopy (SEM)

The porous microstructure of the hydrogel was observed using SEM. The hydrogel was lyophilized to completely remove the water. The samples were imaged using SEM (SEM-Quanta400, Fei, Zlin, Czech Republic) at 20 kV after coating with a thin layer of gold with a Sputter Coater 11430/11428 machine (SPI, Coatesville, PA, USA). All images were analyzed by xT microscope Control software version 4.5.

#### 4.3.6. Cell Culture and MTT Assay

The primary HDLECs were cultured with endothelial cell basal medium MV2. The medium was supplemented with growth medium MV2 supplement mix. Both were purchased from PromoCell (PromoCell, Heidelberg, Germany). HDLECs from passages 6–8 were used for the experiments. For each experiment, the plating density of the cells was 2 × 10^4^ cells/cm^3^ and they were allowed to adhere for 24 h in a humidified CO_2_ incubator maintained at 37 °C and 5% CO_2_. Cells were trypsinized with CTS Versene solution for 5 min and 0.04% trypsin-EDTA for a minute. The cells were then dislodged completely. The trypsin activity was stopped in the culture medium and the whole solution was transferred to a 15 mL conical centrifuge tube. Centrifugation was carried out at 500 g for 5 min. Afterwards, the supernatant was removed and the cells were suspended and counted using the trypan blue assay on a Countess^®^ automated cell counter (Invitrogen, Life Technologies, Carlsbad, CA, USA).

The non-adherent THP-1 cells were cultured in RPMI-1640 medium supplemented with 10% FBS, 1% penicillin-streptomycin, and 10 µL of 0.05 mM β-mercaptoethanol was added in 50 mL of supplemented RPMI-1640. The cells were cultured in a CO_2_ incubator maintained at 37 °C and then sub-cultured and transferred to a 15 mL centrifuge tube followed by centrifugation at 500× *g* for 5 min. Afterwards, the supernatant was removed and the cells were counted by using the trypan blue assay on a Countess^®^ automated cell counter (Invitrogen, Life Technologies, Carlsbad, CA, USA).

Cell viability was assessed using the MTT assay. The MTT assay was based on the conversion of yellow-colored tetrazolium MTT into purple-colored formazan crystals by the mitochondrial reductase enzyme in living cells. The amount of formazan crystals is proportional to the metabolic activity in living cells. Cells were seeded at 10^4^ cells/well into 96-well plates and incubated for 24 h in culture medium. After cell attachment, the culture medium containing 3.9, 7.8, 15.6, 31.3, 62.5, 125, and 250 µg/mL of CBD-β-CD-PM or THC-Me-β-CD or culture medium containing 1.6, 3.1, 6.3, 12.5, 25, 50, and 100 µg/mL of the CBD-THC hydrogel formulations M-1A, M-1B, M-2A, M-2B, M-3A and M-3B, respectively, were added without further extraction. A negative control and a blank hydrogel were run in parallel. Four wells were utilized in each group. They were incubated in a CO_2_ incubator maintained at 37 °C and 5% CO_2_ for 24 h. Subsequently, 100 µL of MTT solution (5 mg/mL) was added to each well and incubated at 37 °C for 2 h. After incubation, the MTT solution was removed. Then, 100 µL of 100% DMSO was added to each well to dissolve the formazan crystals at 37 °C. The absorbance of each well was measured at 570 nm using a microplate reader (BMG LABTECH, Ortenberg, Germany). Cell viability was calculated using the following formula:

$$\mathrm{Viability}\;(\%)=\frac{Absorbance\;of\;sample}{Absorbance\;of\;negative\;control\;}\times 100$$

#### 4.3.7. Collagen Assay

Total soluble collagen in the cell culture supernatants was quantified for an estimation of collagen production in the formulations using the Sircol™ collagen assay kit (Biocolor, Belfast, UK). For these experiments, cells at 10^4^ cells/well were seeded into 96-well plates and incubated at 37 °C for 24 h in the culture medium. The old medium was then removed and replaced with 50 µL fresh medium. An amount of 50 µL each of the CBD-THC hydrogel formulations (M-1A, M-1B, M-2A, M-2B, M-3A and M-3B) was then added and the cells were incubated for another 24 h at 37 °C. Before using the general protocol of the Sircol™ collagen assay, 10 µL of the supernatant was used prior to extraction of collagen. Briefly, 100 µL of 0.1 mg/mL acid pepsin solution was added to the 10 μL of supernatant and incubated with gentle rotation overnight at 4 °C. After incubation, centrifugation was carried out at 3000× *g* for 10 min. Then, the supernatant was collected and stored at −20 °C. Afterwards, the general protocol of the Sircol™ collagen assay was used by adding 100 µL of Sircol™ dye reagent (an anionic dye that reacts specifically with basic collagen side chain groups) into 100 μL of the supernatant, vortexed for 10 min at 25 °C, and centrifuged at 10,000 g for 10 min. The supernatant was removed, and then, the unbound dye was washed using 750 µL of ice-cold acid salt wash reagent and centrifuged at 10,000 g for 10 min. The supernatant was removed, and the remaining pellet was re-dissolved by the addition of 100 µL of alkali reagent. The absorbance was then measured at 556 nm. Fresh medium was used as the blank while concentrations of 0, 10, 25 and 50 μg/mL of the collagen reference were used to prepare standard curves. Four wells were measured in each group.

#### 4.3.8. Tube Formation Assay

The ability of HDLECs to form tubule-like structures when exposed to the formulations was determined by a tube formation assay to estimate the lymphangiogenesis effect of the formulations. Briefly, 24-well plates were coated with ice-cold Matrigel solution and incubated at 37 °C for 30 min to allow the Matrigel to solidify. The 8 × 10^4^ cells/well of HDLECs were suspended in 900 µL of culture medium. Afterwards, the cells were treated using 100 µL of blank hydrogel, 2 µg/mL CBD-β-CD-PM, 2 µg/mL THC-Me-β-CD in sterile water for injection, and 100 µL CBD-THC hydrogel formulations (M-1A, M-1B, M-2A, M-2B, M-3A and M-3B) without extraction. Image progression of HDLECs tube formation was assessed by a Zeiss Axio Observer Z1 microscope and Zeiss Zen 3.8 software (Carl Zeiss Microscopy GmbH, Oberkochen, Germany) at 6 h and 8 h, respectively. The numbers and lengths of the tubes were quantified using the Image J software (NIH, Bethesda, MD, USA). This plugin is an extension of the Angiogenesis Analyzer.

#### 4.3.9. Lipopolysaccharide Stimulation and Anti-Inflammatory Assay

THP-1 cells were seeded in a 48-well plate at a density of 10^6^ cells/well in 200 µL of RPMI-1640 medium. The cells were incubated for 3 h at 37 °C in a 5% CO_2_ incubator. After incubation, 100 µL of lipopolysaccharides (LPS) (1 µg/mL) was added along with 100 µL each of CBD-β-CD-PM (15 µg/mL), THC-Me-β-CD (15 µg/mL), and blank hydrogel and the six CBD-THC hydrogel formulations (M-1A, M-1B, M-2A, M-2B, M-3A, and M-3B [each at 15 µg/mL]), respectively. After that, RPMI-1640 medium was added to obtain final volumes of 500 µL. The samples with LPS were thoroughly mixed. Control wells contained only RPMI-1640 medium. The well plates were incubated at 37 °C in a 5% CO₂ for 24 h. After that, 200 µL of cell supernatants were collected from each well for the ELISA assay.

IL-1 production in the cell culture supernatants was quantified using the Quantikine^®^ human IL-1β immunoassay that was performed according to the manufacturer’s instructions. The microplates provided with the kit were pre-coated with a capture antibody specific for human IL-1β. Briefly, 200 μL each of the standard, control, or sample was added to each well of the microplate, and the plate was covered with an adhesive strip. The microplates were incubated for 2 h at room temperature. After incubation, each well was aspirated and washed three times with 400 μL of wash buffer per well. After the final wash, any remaining wash buffer was removed, and the plate was inverted for dryness. Afterward, 200 μL of human IL-1β conjugate solution was added to each well, covered with a new adhesive strip, and incubated for 1 h at room temperature. The wells were then aspirated and washed three times with 400 μL of wash buffer per well. After the final wash, any remaining wash buffer was removed, and the plate was inverted for dryness. Subsequently, 200 μL of substrate solution was added to each well and incubated for 20 min at room temperature in the dark. After this incubation, 50 μL of stop solution was added to each well to stop the peroxidase reaction that resulted in a color intensity proportional to the concentration of IL-1β. The absorbance was measured at 450 nm using a microplate reader. The absorbance results were quantified using a standard curve.

### 4.4. Statistical Analysis

Statistical analysis was performed using Microsoft Excel. All data are presented as mean ± standard deviation. Comparisons between the untreated and treated groups were analyzed by student *t*-tests. A *p*-value < 0.05 was considered statistically significant.

## 5. Conclusions

The CBD-THC hydrogel formulations demonstrated favorable physicochemical properties, including appropriate particle size, PDI, zeta potential, pH, and shear-thinning behavior. The hydrogel microstructures and pore sizes were suitable for cell growth promotion. Cytotoxicity assessments revealed that THC-Me-β-CD and CBD-β-CD-PM were non-toxic to HDLECs at concentrations below 125 μg/mL and 62.5 μg/mL, respectively. All formulations were non-toxic at CBD-β-CD-PM concentrations below 12.5 μg/mL and THC-Me-β-CD concentrations below 6.25 μg/mL. CBD-THC hydrogels exhibited bioactivity by influencing collagen production, a critical factor in lymphedema treatment. Given that excessive collagen deposition worsens fibrosis and lymphatic dysfunction, formulations with lower concentrations of high MW HA (0.1%) may be preferable for lymphedema management. In contrast, formulations such as M-1B, M-2B, and M-3B, which significantly increased collagen production, may be less suitable due to their potential to exacerbate fibrosis. Angiogenesis assays demonstrated that CBD-THC hydrogels enhanced tube formation in HDLECs, which could aid lymphatic and blood flow, promoting fluid drainage and reducing swelling. The M-1A formulation was particularly effective in inducing angiogenesis, suggesting its potential for lymphatic vessel formation. Notably, 2 μg/mL of THC-Me-β-CD significantly enhanced angiogenesis in vitro. The inflammatory cytokine analysis indicated that CBD-THC hydrogels suppressed inflammatory responses in LPS-activated THP-1 cells. Among the formulations, M-2B exhibited the most potent anti-inflammatory effects, likely due to an optimized CBD-THC ratio. These findings highlight the potential of CBD-THC hydrogels as anti-inflammatory treatments with enhanced efficacy compared to individual compounds. Overall, formulations that promote angiogenesis without excessive collagen production are preferred for lymphedema treatment. Their ability to enhance angiogenesis, improve fluid drainage, and support tissue repair makes them strong candidates for further research and potential clinical application. However, careful monitoring is necessary to ensure these treatments promote lymphatic repair without contributing to fibrosis. Optimizing particle size distribution and morphology will be crucial for developing injectable hydrogels for lymphedema treatment. Additionally, THC-Me-β-CD showed promising angiogenic potential, warranting further investigation. Future studies should focus on validating these findings in vivo and exploring mechanistic pathways to refine targeted therapies. A promising direction is the development of new drugs that modulate key inflammatory pathways involved in lymphedema pathogenesis. In conclusion, CBD-THC hydrogels offer a multifunctional therapeutic strategy for lymphedema by combining angiogenesis promotion, anti-inflammatory effects, and injectable gelation behavior. Their ability to reduce IL-1β, promote tube formation, and regulate collagen production positions them as promising candidates for further research and clinical translation. Future work should emphasize optimizing HA content, assessing dose-dependent effects, and advancing these formulations toward in vivo validation and clinical application.

## Figures and Tables

**Table 1:** Physicochemical properties of the hydrogel formulations and ingredients (mean ± SD, n = 3).

Compositions	Code	L:H Ratio * (g:g)	Size (nm)	PDI	Zeta (mV)	pH	THC (%)	CBD (%)
**Ingredients**	CBD-β-CD-PM	-	223.18 ± 5.30	0.603 ± 0.02	−7.57 ± 1.47	6.71 ± 0.09	-	98 ± 2.2
	THC-Me-β-CD	-	1224.68 ± 239.18	0.791 ± 0.09	−7.96 ± 2.04	6.49 ± 0.10	95 ± 1.5	-
	HA (L)	-	532.08 ± 8.48	0.423 ± 0.03	−37.15 ± 3.18	6.49 ± 0.02	-	-
	HA (H)	-	424.80 ± 25.80	0.925 ± 0.02	−41.60 ± 2.59	6.64 ± 0.06	-	-
**CBD-β-CD-PM** **1 mg/mL**	C-1A	0.05:0.10	230.87 ± 114.70	0.54 ± 0.13	−41.03 ± 3.78	6.53 ± 0.01	-	81.89 ± 1.56
	C-1B	0.05:0.20	246.33 ± 43.76	0.47 ± 0.03	−56.43 ± 4.77	6.38 ± 0.03	-	82.10 ± 2.82
	C-2A	0.10:0.10	143.13 ± 2.33	0.41 ± 0.01	−27.80 ± 4.14	6.47 ± 0.02	-	74.11 ± 2.86
	C-2B	0.10:0.20	182.60 ± 1.93	0.48 ± 0.04	−40.47 ± 5.01	6.37 ± 0.01	-	78.02 ± 2.64
	C-3A	0.20:0.10	153.03 ± 3.05	0.42 ± 0.03	−29.80 ± 2.27	6.49 ± 0.02	-	80.41 ± 5.16
	C-3B	0.20:0.20	184.83 ± 7.66	0.45 ± 0.03	−33.63 ± 3.69	6.43 ± 0.02	-	83.26 ± 1.57
**THC-Me-β-CD 0.5 mg/mL**	T-1A	0.05:0.10	1172.00 ± 161.46	0.88 ± 0.03	−42.40 ± 3.65	6.34 ± 0.02	95.50 ± 2.49	-
	T-1B	0.05:0.20	780.50 ± 122.38	0.72 ± 0.03	−61.63 ± 3.32	6.31 ± 0.02	82.62 ± 1.68	-
	T-2A	0.10:0.10	568.20 ± 73.27	0.60 ± 0.07	−45.17 ± 2.63	6.34 ± 0.08	80.83 ± 2.52	-
	T-2B	0.10:0.20	679.47 ± 29.29	0.68 ± 0.03	−62.63 ± 1.69	6.46 ± 0.06	84.96 ± 2.33	-
	T-3A	0.20:0.10	750.13 ± 102.32	0.76 ± 0.09	−37.27 ± 1.21	6.45 ± 0.02	81.07 ± 2.46	-
	T-3B	0.20:0.20	1467.00 ± 396.19	0.96 ± 0.07	−46.23 ± 0.50	6.37 ± 0.01	96.69 ± 2.47	-
**CBD-β-CD-PM 0.1 mg/mL** **THC-Me-β-CD 0.05 mg/mL**	M-1A	0.05:0.10	456.90 ± 60.36	0.59 ± 0.09	−49.00 ± 4.06	6.43 ± 0.01	73.20 ± 4.14	94.34 ± 1.27
	M-1B	0.05:0.20	545.07 ± 34.11	0.60 ± 0.08	−58.80 ± 1.47	6.54 ± 0.03	94.47 ± 4.22	87.83 ± 3.61
	M-2A	0.10:0.10	302.03 ± 41.10	0.46 ± 0.04	−37.90 ± 1.56	6.46 ± 0.02	76.77 ± 5.25	84.30 ± 1.33
	M-2B	0.10:0.20	441.90 ± 34.37	0.66 ± 0.01	−52.57 ± 3.60	6.48 ± 0.02	92.00 ± 4.46	80.49 ± 4.23
	M-3A	0.20:0.10	308.43 ± 48.79	0.42 ± 0.07	−34.97 ± 1.88	6.53 ± 0.02	96.14 ± 4.59	78.82 ± 0.05
	M-3B	0.20:0.20	357.17 ± 10.10	0.55 ± 0.04	−45.33 ± 1.88	6.46 ± 0.01	72.94 ± 4.12	84.08 ± 0.85

* HA (L): low-molecular-weight hyaluronic acid, HA (H): high-molecular-weight hyaluronic acid. CBD-β-CD-PM is cannabidiol-β-cyclodextrin polymeric micelle. THC-Me-β-CD is tetrahydrocurcumin-methyl-β-cyclodextrin.

**Table 2:** IL-1β levels in cell supernatant of THP-1 cells (mean ± SD, n = 4).

Sample	IL-1β Level (pg)	%IL-1 β Inhibition *
**(-) Control**	3.99 ± 0.15	-
**LPS activated**	68.18 ± 4.66	-
**CBD-β-CD-PM**	4.85 ± 2.01	11.72 ± 1.68
**THC-Me-β-CD**	11.85 ± 2.69	4.53 ± 2.70
**Blank hydrogel**	8.35 ± 3.89	103.93 ± 0.70
**M-1A**	22.37 ± 1.92	57.46 ± 8.76
**M-1B**	12.03 ± 2.60	64.51 ± 3.14
**M-2A**	12.07 ± 2.71	66.18 ± 1.92
**M-2B**	8.52 ± 0.53	68.97 ± 3.70
**M-3A**	10.92 ± 3.22	63.12 ± 12.66
**M-3B**	8.09 ± 0.85	56.86 ± 6.10

* The percent inhibition were calculated from inactivated THP-1, activated THP-1 and activated THP-1 treated with samples. %IL-1β inhibition = $\frac{Abs\;of\;activated\;THP-1-Abs\;of\;activated\;THP-1\;treated\;with\;samples}{Abs\;of\;activated\;THP-1-Abs\;of\;inactivated\;THP-1}\times 100.$

**Table 3:** Functional ingredients of the hydrogel formulations.

Ingredients	Function
HA	Gelling agent
Chlorobutanol	Preservative
Propylene glycol	Co-dispersion medium
CBD-β-CD-PM	Active ingredient
THC-Me-β-CD	Active ingredient
Sterile water for injection	Dispersion medium

**Table 4:** Compositions of the hydrogel formulations.

Hydrogel Formulation Code	HA (L) (mg)	HA (H) (mg)	CBD-β-CD-PM (mg)	THC-Me-β-CD (mg)
C-1A	50	100	100	-
M-1A	50	100	10	5
T-1A	50	100	-	50
C-1B	50	200	100	-
M-1B	50	200	10	5
T-1B	50	200	-	50
C-2A	100	100	100	-
M-2A	100	100	10	5
T-2A	100	100	-	50
C-2B	100	200	100	-
M-2B	100	200	10	5
T-2B	100	200	-	50
C-3A	200	100	100	-
M-3A	200	100	10	5
T-3A	200	100	-	50
C-3B	200	200	100	-
M-3B	200	200	10	5
T-3B	200	200	-	50

Note: All formulations contain 0.5% chlorobutanol. HA (L): low-molecular-weight hyaluronic acid, HA (H): high-molecular-weight hyaluronic acid.

## Data Availability

The original contributions presented in this study are included in the article. Further inquiries can be directed to the corresponding author.

## Abbreviations

CBD	Cannabidiol
THC	Tetrahydrocurcumin
CBD-β-CD-PM	Cannabidiol-β-cyclodextrin inclusion poloxamer-based micelle
THC-Me-β-CD	Tetrahydrocurcumin-methyl-β-cyclodextrin inclusion complex
PG	Propylene glycol
HA	Hyaluronic acid
VEGFR-3	Vascular endothelial growth factor receptor 3
VEGF-C	Vascular endothelial growth factor C
Foxc2	Forkhead box C2 transcription factor
SOX18	SRY-box transcription factor 18
HDLECs	Human dermal lymphatic endothelial cells
LECs	Lymphatic endothelial cells
SEM	Scanning electron microscopy
